# Chorea as the only presenting clinical feature of rheumatic fever: a case report

**DOI:** 10.1097/MS9.0000000000001798

**Published:** 2024-02-09

**Authors:** Santosh Thapa, Ujwal Raut, Garima Shrestha, Sandesh Shah, Mangal Bahadur Helmu

**Affiliations:** a B. P. Koirala Institute of Health Sciences, Dharan; b KIST Medical College and Teaching Hospital, Lalitpur; c Department of Pediatrics, National Academy of Medical Sciences, Kathmandu, Nepal

**Keywords:** acute rheumatic fever (ARF), Milkmaid’s grip, subclinical carditis, Sydenham’s chorea (SC)

## Abstract

**Introduction and importance:**

Sydenham’s chorea (SC), a major neurological manifestation of acute rheumatic fever (ARF), is commonly seen in young children and adolescents. It is characterized by rapid, unpredictable, involuntary, and nonpatterned contractions affecting mostly distal limbs. It can also be associated with clinical or subclinical carditis. SC has been reported as a major manifestation in only 3.87% cases of acute rheumatic fever in Nepal.

**Case presentation:**

The authors report a case of a 12-year-old boy with abnormal movement of his right hand and unsteady gait for 12 days. On examination, he had an abnormal hand grip with difficulty maintaining a tetanic contraction (Milkmaid’s grip). Laboratory investigations revealed increased anti-Streptolysin O titre and erythrocyte sedimentation rate. Echocardiography revealed subclinical carditis. After thorough clinical examination and pertinent investigations, the final diagnosis of ARF with SC was made.

**Clinical discussion:**

SC is a major clinical feature of rheumatic fever according to the revised Jones criteria. It is related to a previous Group A β-haemolytic *Streptococcus pyogenes* (GABHS) infection. Approximately 50–65% of the patients with rheumatic fever later develop clinically detectable carditis. Although a self-limiting condition, it might need treatment with antiepileptics, neuroleptics, and phenothiazines.

**Conclusion:**

Any child presenting with a movement disorder should also be considered for SC, necessitating additional testing, including a cardiovascular assessment. It needs to be distinguished from other causes of movement disorders as well as psychiatric conditions. Treatment is necessary for moderate to severe chorea that interfere with daily activities. Compliance with subsequent antibiotic prophylaxis is essential for avoiding future cardiac complications.

## Introduction

Highlights

- A 12-year-old male presented with abnormal movement of the right hand.
- On general examination, jerky, nonpatterned, and involuntary movements of his right upper limb were noticed with variable speed, timing, and direction.
- During motor examinations, he had abnormal hand grip with difficulty maintaining a tetanic contraction with his hands, “Milkmaid’s sign”.
- Laboratory evaluation revealed an elevated anti-Streptolysin O (ASO) titre. Echocardiogram showed thickened anterior mitral leaflet, slightly restricted motion of posterior mitral leaflet, and pseudo prolapse of the anterior mitral leaflet with mild eccentric mitral regurgitation along with trivial tricuspid regurgitation, trivial aortic regurgitation and trivial pulmonary regurgitation.
- The final diagnosis of acute rheumatic fever with Sydenham’s chorea was made.

Sydenham’s chorea (SC), often known as chorea minor, is a condition that causes jerky, uncoordinated movements, particularly in the hands and face^1^. According to a study, 3.87% of acute rheumatic fever (ARF) patients in Nepal have SC, which is far less common than 5–38% as reported by WHO^1^. The most frequent clinical presentation of ARF when it manifests as a neurological illness is SC without joint symptoms. Carditis, if present, is typically subclinical^2^. In many instances, other symptoms of rheumatic fever may subside when SC appears as it is a delayed manifestation^3^. The patient might not have a history of ARF, and a prior streptococcal infection is not usually possible to prove. Infections can be subclinical and frequently appear 1–6 months before neurologic symptoms appear^1^. The chorea often develops subacutely and is bilateral, although 20–30% of cases may be unilateral, known as hemichorea. Although the patient’s mental state is usually normal, SC is frequently mistaken as a mental or psychiatric illness^4^. Following a Streptococcal infection, the cross-reaction between Streptococcal antigens and human tissue antigens through antigen mimicry leads the immune system target the basal ganglia of the brain. This inflammatory process results in Sydenham chorea^5^. Rheumatic heart disease (RHD) is caused by damage to the heart valves during episodes of ARF following group A streptococcal infection^6^. In comparison to the global prevalence and mortality data of 0.54% and 0.54%, respectively, the prevalence of rheumatic heart disease was 0.57%, and the total death rate from rheumatic heart disease was 1.45% in Nepal^7^. We report a case of a patient with SC as the only presenting clinical feature of rheumatic fever. The primary learning goal of our shown instance is the potential for the single presentation of the given diagnosis.

Guidelines: SCARE 2023 paper^8^, Supplemental Digital Content 1, http://links.lww.com/MS9/A375.

This case has been reported in line with the SCARE 2023 criteria.

## Case report

### Patient information

#### Demographics and presentation

A 12-year-old male presented with the complaint of abnormal movement of the right hand for 12 days. According to the informant, he was in a usual state of health when he developed abnormal jerky movement of his right hand which was progressive, and continuous. The hand movement could not be suppressed voluntarily and it aggravated via stress and awake state and relieved during sleep. Over time, the condition progressed to include difficulty in walking and writing. On further inquiry, there was no history of waxing and waning and diurnal variation. There was no history of any joint pain and swelling, photosensitivity, fatigue, and rash. There was also no history of fever, headache, vomiting, and recurrent throat infections. There was no any indication of history of heat intolerance, significant weight loss, palpitation, and change in mood. Furthermore, there was no history of vomiting, convulsion, blurring of vision, yellowish discoloration of the sclera, and decreased urine output. Finally, there was no history of falls or trauma.

#### Past medical and surgical history

His past medical and surgical histories were insignificant.

#### Family history

There was no history of behavioural or neuropsychiatric disorders in the family. There was no history of consanguineous marriage in the family.

#### Developmental and immunization history

His developmental history and immunization records were as per age.

#### Birth history

He was delivered via spontaneous, normal vaginal delivery at term and the perinatal period was uneventful. There was no history of yellowish discoloration of the body.

#### Drug and allergy history

He had no history of drug intake or allergies.

### Clinical findings

On presentation, the patient was alert, afebrile, and well-oriented to time, place, and person. He was anxious and restless with involuntary movements and unable to stand without support. His vital signs were stable. On general examination, jerky, nonpatterned, involuntary, and random movements of his right upper limb were noticed with variable speed, timing, and direction (SDC Video 1, Supplemental Digital Content 2, http://links.lww.com/MS9/A376). A facial grimace was also noticed. On neurological examination, his higher mental functions, cranial nerves, and sensory examinations were within normal limits. However, during motor examinations, he had abnormal hand grip with difficulty maintaining a tetanic contraction with his hands, “Milkmaid’s sign” (SDC Video 2, Supplemental Digital Content 3, http://links.lww.com/MS9/A377). Deep tendon reflexes were normal and symmetrical on both sides, and his toes were down-going. Cerebellar signs could not be assessed due to the jerky movements. However, his gait was unstable and he ambulated with assistance to avoid falling. No other manifestation of RF was detected. Examination of the cardiovascular system revealed normal heart sounds and no audible murmurs. Ophthalmologic examination revealed no Kayser–Fleischer (KF) rings. On examination, there were no signs of meningeal irritation. The rest of the physical examination was otherwise unremarkable.

### Diagnostic assessment and interpretation

**Figure 1:**
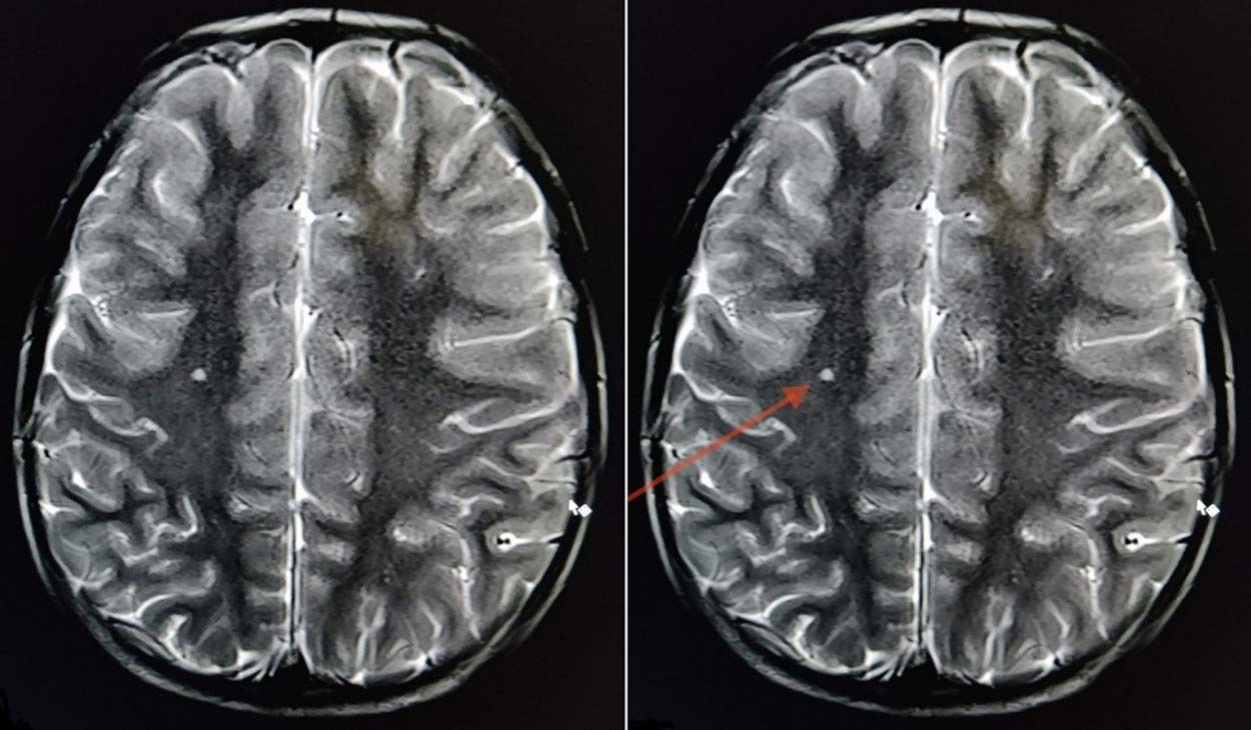
T2/FLAIR high signal intensity foci in right parietal lobe (left unmarked, right marked); features suggestive of non-specific demyelination (red arrow) and rest of the brain showing normal morphology with normal parenchymal signal intensity with no evidence of brain oedema, recent haemorrhage or infarction.

Laboratory evaluation revealed an anti-Streptolysin O (ASO) titre (quantitative) of 222 IU/ml (normal: <200.0 IU/ml) and an erythrocyte sedimentation rate (ESR) of 24.0 mm/h (normal: 0–22 mm/h) (Table 1). The basic metabolic panel, liver function tests, urine tests, and blood culture tests were all unremarkable. Anti-nuclear antibody report was found to be negative. MRI Brain revealed features suggestive of non-specific foci of demyelination (Fig. 1).

**Table 1:** Patient information and his laboratory and radiological findings

Patient age/sex: 12 years/m				
S. no.	Test	Result	Unit	Reference range
1.	ASO titre-quantitative	222	IU/ml	<200.0
2.	ESR (Wintrobe method)	24.0	mm/h	0–22
3.	ANA	9.76	AU/ml	Negative: <40 Positive: >40
4.	Echocardiogram	i Thickened anterior mitral leaflet (thickness 3.5 mm) ii Slightly restricted motion of posterior mitral leaflet iii Pseudo prolapse of anterior mitral leaflet with mild eccentric mitral regurgitation iv Trivial tricuspid regurgitation (Gradient: 17 mmHg) v Trivial aortic regurgitation vi Trivial pulmonary regurgitation		
5.	MRI brain	T2/FLAIR high intensity foci in right parietal lobe; features suggestive of non-specific demyelination		

ANA, anti-nuclear antibody; ASO, anti-Streptolysin O; ESR, erythrocyte sedimentation rate.

The patient was admitted for further evaluation. Electrocardiogram was normal. Echocardiogram was done which showed thickened anterior mitral leaflet (thickness of 3.5 mm), slightly restricted motion of posterior mitral leaflet, and pseudo prolapse of the anterior mitral leaflet with mild eccentric mitral regurgitation along with trivial tricuspid regurgitation (gradient: 17 mmHg), trivial aortic regurgitation and trivial pulmonary regurgitation. The biventricular function (left ventricular ejection fraction= 65%) and chambers were all found to be normal and no clots, vegetation, or pericardial effusion could be appreciated. The final diagnosis of ARF with SC was made.

### Intervention and follow-up

He was started on haloperidol, 0.5 mg per day in two divided doses. The dose was gradually up-titrated during his hospital stay to 1 mg per day in two divided doses. No adverse reactions were noted during this period. Secondary prophylaxis with 1.2 million units of IM benzathine penicillin was given. He was discharged on 14th day of admission with oral penicillin V 250 mg twice daily along with a maintenance dose of haloperidol. The patient followed up after a month. His chorea had improved to some degree. He was compliant with the prescribed penicillin.

## Discussion

SC is a movement disorder that is characterized by involuntary choreiform movements that include facial grimacing, hypotonia, muscle weakness, gait disturbances, difficulty writing and speaking, tics, and dysarthria. It is often associated with psychological symptoms like emotional lability, obsessive-compulsiveness, anxiety, and depression. It is related to a previous Group A β-haemolytic *Streptococcus pyogenes* infection (GABHS)^9^. Although it is widely considered to be an autoimmune disorder, the exact pathophysiology is still not well established. It is believed that antibodies against GABHS cross-react with the neurons of the basal ganglia. These anti-basal ganglia antibodies react with the surface of neuronal cells and signal the induction of calcium calmodulin-dependent protein kinase II. Thus the tyrosine hydroxylase level becomes elevated leading to the release of dopamines, which in turn leads to the movement disorder. Many pathological studies have shown neuronal loss, cytoplasmic and nuclear cell changes, gliosis, endothelial swelling, perivascular round cell infiltration, and petechial haemorrhages within the cerebral cortex, basal ganglia, and thalamus^10^.

In most people, SC is bilateral. It can, however, present largely unilaterally in some patients, necessitating additional neurological testing to rule out any additional potential reasons for the movement issue. Drug reactions, Wilson disease, Systemic Lupus Erythematosus, and Huntington chorea should all be ruled out. It is also important to distinguish the motions from tics, athetosis, conversion reaction, and hyperkinesis^11^. Four separate motor tests are routinely performed during a physical examination: the spooning test, the touchdown test, the milkmaid’s grip test, and the tongue darting test. The characteristic movements of SC are often revealed by these four manoeuvres. On examination, there should typically be no sensory loss. Due to the chorea in the muscles that make movement possible, gait is frequently described as unstable^4^. Our patient also demonstrated the milkmaid’s grip, and his gait was also unstable.

The goal of a diagnostic examination should be to identify acute rheumatic fever. Along with a cardiac assessment, testing should be done to look for a GABHS infection. Electrocardiograms and echocardiograms should be part of cardiac testing. Blood tests for streptococcal antibodies, such as ASO and anti-deoxyribonuclease B, are frequently performed. Serum ASO titres are typically elevated and peak at about 3–5 weeks post-infection, declining thereafter^4^. Although two-thirds of cases report elevated ASO titres, these are not helpful in the diagnosis of SC^10^. The ASO titre when measured was found to be elevated along with ESR in our patient.

Neurologic testing such as a lumbar puncture, brain MRI, or computed tomography scan of the skull can be helpful to rule out additional neurologic and psychiatric mimics^4^. SC may be associated with MRI abnormalities of non-specific hyperintense white matter lesions. These anomalies might result from the inflammatory process linked to clinical signs that last for a longer time. Our patient’s MRI also revealed similar non-specific findings. Immunomodulatory treatments could be successful for SC due to the autoimmune component in the disease’s aetiology. Comprehensive research is required to explain the mechanisms underlying the MRI findings and the pathogenesis of SC^10^.

SC is a major clinical feature of rheumatic fever according to the revised Jones criteria, 2015^11^. Also, ~50–65% of the patients with rheumatic fever later develop clinically detectable carditis (inflammation of the heart valve leaflets leading to valvular regurgitation). Carditis can also be in a milder form which may be subclinical in 30% of the cases and is detectable only by echocardiography and not by auscultation with a stethoscope^12^. The risk of developing severe chronic rheumatic heart disease, which is linked to an increased risk of heart failure, infective endocarditis, pregnancy complications, stroke, arrhythmias, and premature death, is highest in people who have severe carditis during the initial episode of rheumatic fever or who have recurrences of the illness^13^.

RHD is considered to be one of the most common acquired heart diseases, mostly affecting children and young adults in developing countries. It represents about 15–20% of all the patients with heart failure living in endemic countries^14^. It is estimated that up to 40% of RHD patients do not have a preceding history of diagnosed rheumatic fever episodes^13^. Up to 60% of the patients with isolated chorea findings are estimated to have residual RHD^13^.

With a mean duration of 2–4 months, SC is a self-limiting condition. For people whose chorea is not mild, however, it requires treatment. Antiepileptics, neuroleptics, and phenothiazines have been observed to lessen the abnormal movements by influencing the dopaminergic or alpha-aminobutyric acid pathways. Due to the autoimmune aetiology, intravenous immunoglobulin, plasma exchange, and corticosteroids successfully suppress uncontrollable movements^15^. In 15–30% of patients, chorea does recur. Although delayed cases have occurred as late as 10 years, this typically happens within 1–3 years. Those who are not receiving continuous antibiotic therapy are more likely to experience relapses. The likelihood of developing chronic rheumatic heart disease does increase with recurrent GABHS infection and SC^4^.

Regardless of whether isolated SC or cases with cardiac disease are found, secondary prophylaxis is recommended in all patients of ARF. To prevent chorea relapses and a worsening of the valve disease, subsequent adherence to secondary prophylaxis is extremely essential^13^. In this regard, injectable penicillin G benzathine administered every 3–4 weeks is the recommended approach. Daily oral penicillin V is also regarded as an alternative regimen for patients or families who object to the standard injection schedule^16^. Our patient opted for an oral regimen.

Expert opinion and the observation that recurrences are extremely rare beyond the age of 21 or more than 10 years after the initial acute rheumatic fever episode are the basis for recommendations for the length of secondary prophylaxis with penicillin. Despite regional variations in practice, the WHO also advises that prophylaxis for individuals with carditis be continued for at least 10 years after diagnosis. In people with severe rheumatic heart disease, particularly those who have undergone cardiac surgery or experienced recurrences, prophylaxis should be continued for a longer period^13^.

## Conclusion

Chorea in childhood that has recently appeared should be suspected of SC. It is possible to misinterpret SC, an unusual manifestation of rheumatic fever, for the symptoms of a fidgety child or a psychiatric manifestation. When hemichorea is present in children with normal neuroimaging, SC should be considered. Due to the likelihood of subclinical valvular lesions, all patients with suspected SC require prompt cardiological studies. The results of these investigations will guide patient management and prognosis. Early diagnosis is crucial because preventive antibiotics and immediate treatment might lessen heart damage and improve symptoms.

## Ethical approval

None.

## Consent

Written informed consent was obtained from the patient’s parents for publication of this case report and accompanying images and videos. A copy of the written consent is available for review by the Editor-in-Chief of this journal on request.

## Source of funding

There is no funding for this research.

## Author contribution

S.T.: conceptualization, methodology, writing—original draft preparation, software, project administration. U.R.: data curation, writing—original draft preparation, software. G.S.: visualization, writing—reviewing and editing. S.S.: supervision, visualization. M.B.H.: validation, supervision, conceptualization.

## Conflicts of interest disclosure

None.

## Research registration unique identifying number (UIN)

1. Name of the registry: Not applicable.
2. Unique identifying number or registration ID: Not applicable.
3. Hyperlink to the specific registration (must be publicly accessible and will be checked): Not applicable.

## Guarantor

Mangal Bahadur Helmu.

## Data availability statement

I confirm that any datasets generated during and/or analyzed during the current study are publicly available.

## Provenance and peer review

Not commissioned, externally peer-reviewed.

## Supplementary Material
