# Supplementary material for: Chorea as the only presenting clinical feature of rheumatic fever: a case report
Source: Ann Med Surg (Lond). 2024 Feb 9;86(4):2162–6. doi: 10.1097/MS9.0000000000001798 (PMC10990359; doi:10.1097/MS9.0000000000001798)
Supplement: Supplementary file 1 [file ms9-86-2162-s001.pdf]

SCARE Checklist			
**Topic**	**Item**	**Checklist item description**	**Page Number**
**Title**	**1**	The words “case report” and the area of focus should appear in the title (e.g. presentation, diagnosis, surgical technique or device or outcome).	1
**Key Words**	**2**	3 to 6 key words that identify areas covered in this case report (include "case report" as one of the keywords).	1
**Abstract**	**3a**	Introduction—What is unique or educational about the case? What does it add to the surgical literature? Why is this important?	1
	**3b**	The patient's main concerns and important clinical findings.	
	**3c**	The main diagnoses, therapeutics interventions, and outcomes.	
	**3d**	Conclusion — what are the “take-away” lessons from this case?	
**Introduction**	**4**	A summary of why this case is unique or educational with reference to the relevant surgical literature and current standard of care (with references, 1-2 paragraphs). Nature of the institution in which the patient was managed; academic, community or private practice setting?	1-2
**Patient Information**	**5a**	De-identified demographic and other patient specific information including age, sex, ethnicity, occupation and other useful pertinent information e.g. BMI and hand dominance.	2
	**5b**	Presentation including presenting complaint and symptoms of the patient as well as the mode of presentation e.g. brought in by ambulance or walked into Emergency room or referred by family physician.	
	**5c**	Past medical and surgical history and relevant outcomes from interventions	
	**5d**	Drug history, family history including any relevant genetic information, and psychosocial history including smoking status and where relevant accommodation type, walking aids, etc.	
**Clinical Findings**	**6**	Describe the relevant physical examination and other significant clinical findings (include clinical photographs where relevant and where consent has been given).	2-3

**Timeline**	7	Inclusion of data which allows readers to establish the sequence and order of events in the patient's history and presentation (using a table or figure if this helps). Delay from presentation to intervention should be reported.	N/A
**Diagnostic Assessment**	**8a**	Diagnostic methods (physical exam, laboratory testing, radiological imaging, histopathology etc).	3
	**8b**	Diagnostic challenges (access, financial, cultural).	
	**8c**	Diagnostic reasoning including other diagnoses considered	
	**8d**	Prognostic characteristics when applicable (e.g. tumour staging). Include relevant radiological or histopathological images in this section (the latter may sometimes be better placed in section 9).	
**Therapeutic Intervention**	**9a**	Pre-intervention considerations e.g. Patient optimisation: measures taken prior to surgery or other intervention e.g. treating hypothermia/hypovolaemia/hypotension in a burns patient, ICU care for sepsis, dealing with anticoagulation/other medications, etc	3
	**9b**	Types of intervention(s) deployed and reasoning behind treatment offered (pharmacologic, surgical, physiotherapy, psychological, preventive) and concurrent treatments (antibiotics, analgesia, anti-emetics, nil by mouth, VTE prophylaxis, etc). Medical devices should have manufacturer and model specifically mentioned.	
	**9c**	Peri-intervention considerations - administration of intervention (what, where, when and how was it done, including for surgery; anaesthesia, patient position, use of tourniquet and other relevant equipment, prep used, sutures, devices, surgical stage (1 or 2 stage, etc). Pharmacological therapies should include formulation, dosage, strength, route, duration, etc).	
	**9d**	Who performed the procedure - operator experience (position on the learning curve for the technique if established, specialisation and prior relevant training).	
	**9e**	Any changes in the interventions with rationale. Include intra-operative photographs and/or video or relevant histopathology in this section. Degree of novelty for a surgical technique/device should be mentioned e.g. "first in-human".	
	**9f**	Post-intervention considerations e.g. post-operative instructions and place of care.	
**Follow-up and Outcomes**	**10a**	Clinician assessed and patient-reported outcomes (when appropriate) should be stated with inclusion of the time periods at which assessed. Relevant photographs/radiological images should provided e.g. 12 month follow-up.	3

	**10b**	Important follow-up measures - diagnostic and other test results. Future surveillance requirements - e.g. imaging surveillance of endovascular aneurysm repair (EVAR) or clinical exam/ultrasound of regional lymph nodes for skin cancer.	
	**10c**	Where relevant - intervention adherence and tolerability (how was this assessed).	
	**10d**	Complications and adverse or unanticipated events. Described in detail and ideally categorised in accordance with the Clavien-Dindo Classification. How they were prevented, diagnosed and managed. Blood loss, operative time, wound complications, re-exploration/revision surgery, 30-day post-op and long-term morbidity/mortality may need to be specified.	
**Discussion**	**11a**	Strengths, weaknesses and limitations in your approach to this case. For new techniques or implants - contraindications and alternatives, potential risks and possible complications if applied to a larger population. If relevant, has the case been reported to the relevant national agency or pharmaceutical company (e.g. an adverse reaction to a device).	3-5
	**11b**	Discussion of the relevant literature, implications for clinical practice guidelines and any relevant hypothesis generation.	
	**11c**	The rationale for your conclusions.	
	**11d**	The primary "take-away" lessons from this case report.	
**Patient Perspective**	**12**	When appropriate the patient should share their perspective on the treatments they received.	N/A
**Informed Consent**	**13**	Did the patient give informed consent for publication? Please provide if requested by the journal/editor. If not given by the patient, explain why e.g. death of patient and consent provided by next of kin or if patient/family untraceable then document efforts to trace them and who within the hospital is acting as a guarantor of the case report.	Title page
**Additional Information**	**14**	Conflicts of Interest, sources of funding, institutional review board or ethical committee approval where required.	5
